# Trends in COVID-19 Incidence After Implementation of Mitigation Measures — Arizona, January 22–August 7, 2020

**DOI:** 10.15585/mmwr.mm6940e3

**Published:** 2020-10-09

**Authors:** M. Shayne Gallaway, Jessica Rigler, Susan Robinson, Kristen Herrick, Eugene Livar, Kenneth K. Komatsu, Shane Brady, Jennifer Cunico, Cara M. Christ

**Affiliations:** ^1^Arizona Department of State Health Services; ^2^CDC COVID-19 Response Team.

*On October 6, 2020, this report was posted online as an *MMWR *Early Release.*

Mitigating the spread of SARS-CoV-2, the virus that causes coronavirus disease 2019 (COVID-19), requires individual, community, and state public health actions to prevent person-to-person transmission. Community mitigation measures can help slow the spread of COVID-19; these measures include wearing masks, social distancing, reducing the number and size of large gatherings, pausing operation of businesses where maintaining social distancing is challenging, working from or staying at home, and implementing certain workplace and educational institution controls (*1*–*4*). The Arizona Department of Health Services’ (ADHS) recommendations for mitigating exposure to SARS-CoV-2 were informed by continual monitoring of patient demographics, SARS-CoV-2 community spread, and the pandemic’s impacts on hospitals. To assess the effect of mitigation strategies in Arizona, the numbers of daily COVID-19 cases and 7-day moving averages during January 22–August 7, 2020, relative to implementation of enhanced community mitigation measures, were examined. The average number of daily cases increased approximately 151%, from 808 on June 1, 2020 to 2,026 on June 15, 2020 (after stay-at-home order lifted), necessitating increased preventive measures. On June 17, local officials began implementing and enforcing mask wearing (via county and city mandates),* affecting approximately 85% of the state population. Statewide mitigation measures included limitation of public events; closures of bars, gyms, movie theaters, and water parks; reduced restaurant dine-in capacity; and voluntary resident action to stay at home and wear masks (when and where not mandated). The number of COVID-19 cases in Arizona peaked during June 29–July 2, stabilized during July 3–July 12, and further declined by approximately 75% during July 13–August 7. Widespread implementation and enforcement of sustained community mitigation measures informed by state and local officials’ continual data monitoring and collaboration can help prevent transmission of SARS-CoV-2 and decrease the numbers of COVID-19 cases.

ADHS supports surveillance and investigation efforts of local public health departments, compiles surveillance and investigation information across counties, and provides infrastructure statewide to support infectious disease surveillance. Data on laboratory-confirmed and probable (*5*) COVID-19 cases (based on the Council of State and Territorial Epidemiologists case definitions)^†^ were collected in the centralized Medical Electronic Disease Surveillance Intelligence System (MEDSIS),^§^ which is used by state, tribal, and county public health agencies to report human-based diseases in Arizona. Information was submitted to or entered into MEDSIS by health care providers, laboratories, local health departments, tribal entities, and ADHS. Multiple laboratory tests submitted for a single patient were combined into a single record. Specimen collection date was used for confirmed cases, and symptom onset date was used for probable cases.

Temporal trends were examined by comparing the number of daily COVID-19 cases (as of September 1)^¶^ and 7-day moving averages before, during, and after implementation of enhanced community mitigation measures, defined as the following: limitations on persons’ time away from their place of residence except for essential activities; certain business closures and service limitations (e.g., occupancy limitations, curbside pickup, and delivery of goods); enhanced sanitation practices**; social distancing, employee mask wearing, and symptom screenings for all businesses operating a physical location; limitations on the occurrence and size of public events; and local mandates enforcing mask use. The 7-day moving average was calculated after the cumulative case count exceeded 20 cases and is presented to describe COVID-19 trends.

On March 11, 2020, Arizona declared a public health state of emergency to prepare for, prevent, respond to, and mitigate the spread of SARS-CoV-2. Additional guidance was provided to local officials, businesses, communities, and individual persons to implement social distancing and close schools statewide (March 15); postpone and limit large gatherings to fewer than 50 persons; recommend telework options; restrict access to congregate settings; require restaurants to provide dine-out options only; and close all bars, gyms, and movie theaters in counties with confirmed COVID-19 cases (March 19) (Table). Based on Arizona data and CDC guidance (*1*,*2*), ADHS also recommended limiting persons’ time away from their place of residence except for essential activities (i.e., stay-at-home order, “Stay Home, Stay Healthy, Stay Connected”)^††^ (March 31).

**TABLE:** Public policies to implement and enforce COVID-19 community mitigation measures and dates of issue/reissue* — Arizona, March 11–August 7, 2020

Mitigation measure	Date of issue/reissue
**Declaration of emergency**	Mar 11
**School closure (on-site learning)**	Mar 15
**Limits on senior living facilities visitation**	Mar 19
**Expanded availability and coverage for telemedicine for persons, pets, and animals**	Mar 25, Apr 1
**Deferred requirements to renew driver license**	Mar 20
**Stay-at-home order**	Mar 30–May 15
**Business/Service closures**	
Bars	Mar 19, Jun 29, Jul 23
Movie theaters	Mar 19, Jun 29, Jul 23
Indoor gyms and fitness clubs	Mar 19, Jun 29, Jul 23
Restaurants, on-site dining	Mar 19
Pools	Mar 19
Water parks and recreational tubing facilities	Jun 29, Jul 23
**Business/Service limits (requirements)**	
All businesses operating a physical location (enhanced sanitation,^†^ social distancing, employee mask wearing, symptom screenings)	Jun 17
Retail (limited capacity, social distancing, enhanced sanitation)	Apr 29
Barbers and cosmetologists (employee mask wearing, spaced appointments, enhanced sanitation)	May 4
Restaurants (social distancing, limited capacity, employee mask wearing, patron mask wearing [when not eating or drinking], employee screening, enhanced sanitation)	May 4, Jul 9
Public pools (e.g., at hotels; limited capacity)	Jun 29, Jul 23
Private pools in public areas (e.g., multihousing complexes; limited capacity)	Jun 29, Jul 23
Public events (<50 persons)	Mar 15, Jun 29, Jul 23
**Wearing masks (mandatory)**	
Local officials able to mandate and enforce wearing masks	Jun 17
Yuma County	Jun 18
Maricopa County	Jun 19
Pima County	Jun 19
Santa Cruz County	Jun 19
Coconino County	Jun 20
>40 other cities/tribal communities	Jun 17–25^§^

**Abbreviation:** COVID-19 = coronavirus disease 2019.

* Issue dates are the dates the issuing official signed the order implementing the mandatory mitigation measure. In some instances, mitigation measures were effective either immediately or within 1 to 3 days of issue. https://www.azdhs.gov/preparedness/epidemiology-disease-control/infectious-disease-epidemiology/index.php#novel-coronavirus-admin-orders; https://azgovernor.gov/executive-orders.

^†^ Based on guidance from the Arizona Department of Health Services, CDC, Department of Labor, and Occupational Safety and Health Administration (OSHA) to limit and mitigate the spread of COVID-19 including promoting healthy hygiene practices; and intensifying cleaning, disinfection and ventilation practices.

^§^ Other tribal communities with mask mandates (issued June 18–23) included Fort McDowell Yavapai Nation, Gila River Indian Community, Navajo Nation, Salt-River Pima-Maricopa Indian Community, Tohono O’Odham Nation. Other cities with mask mandates (issued June 17–25) included Avondale, Bisbee, Buckeye, Carefree, Casa Grande, Chandler, Clarkdale, Clifton, Coolidge, Cottonwood, Douglas, Flagstaff, Fountain Hills, Gila Bend, Gilbert, Glendale, Globe, Goodyear, Guadalupe, Jerome, Kingman, Litchfield Park, Mammoth, Mesa, Miami, Nogales, Oro Valley, Paradise Valley, Payson, Peoria, Phoenix, San Luis, Sedona, Scottsdale, Somerton, Superior, Surprise, Tempe, Tolleson, Tucson, Youngtown, Yuma. Several other tribal communities and cities encouraged but did not mandate wearing masks.

During April 1–May 15, the 7-day moving average of daily cases ranged from 154 to 443 (Figure). During April 29–May 11, Arizona initiated a phased approach for retail shops and stores, cosmetologists, and barbers to reopen and operate, and for restaurants to resume dine-in services; the stay-at-home order ended May 15.

**FIGURE:**
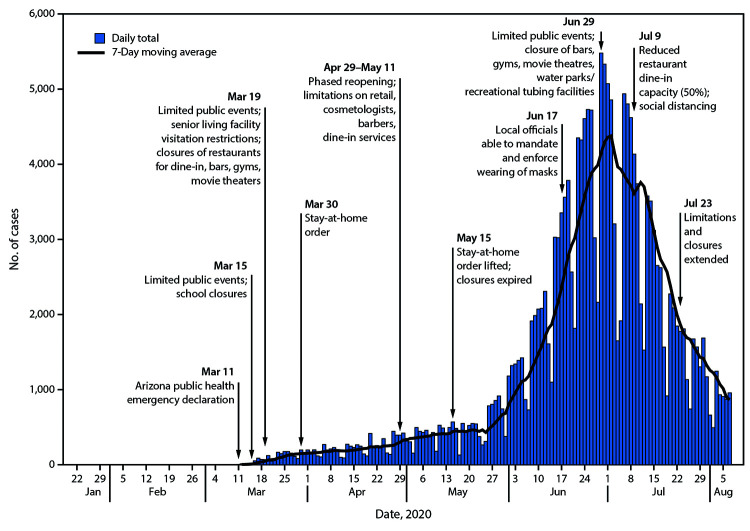
Selected community mitigation measures* and COVID-19 case counts^†^ and 7-day moving averages^§^ — Arizona, January 22–August 7, 2020 **Abbreviation:** COVID-19 = coronavirus disease 2019. * Issue dates are the dates the issuing official signed the order implementing the mandatory mitigation measure. In some instances, mitigation measures were effective either immediately or within 1 to 3 days of issue. https://www.azdhs.gov/preparedness/epidemiology-disease-control/infectious-disease-epidemiology/index.php#novel-coronavirus-admin-orders; https://azgovernor.gov/executive-orders. ^†^ As of September 1, 2020. Specimen collection date was used for confirmed cases, and symptom onset date was used for probable cases. ^§^ Plotting of 7-day moving average began when cumulative case count exceeded 20 cases.

Average daily cases increased 151% from June 1 (808) to June 15 (2,026), necessitating an increased focus on preventive measures by businesses, communities, and individual persons. Updated guidance from state officials provided local governments the authority to implement mask policies (June 17) and enforcement measures tailored to local public health needs (local policies were applicable to approximately 85% of the total Arizona population). Before June 17, mask wearing had not been widely mandated or enforced. Arizona limited organized public events to fewer than 50 persons (with some exceptions); closed bars, gyms, movie theaters, and water parks and recreational tubing facilities (June 29); and limited restaurants’ indoor dining to <50% capacity, with at least 6 feet of separation between patrons (July 9). The 7-day moving average of daily cases peaked during June 29–July 2 (range = 4,148–4,377), stabilized during July 3–12 (range = 3,609–4,160), and subsequently decreased 75% from July 13 (3,506) to August 7 (867). Mitigation measures put in place in June were extended through August to further limit transmission.

## Discussion

Quantitative data on the effectiveness of community mitigation measures at suppressing the spread of COVID-19 are limited. The primary goal of implementing widespread enhanced mitigation measures in Arizona was to protect and save lives and maintain capacity in the health care system. A combination of voluntary and enforceable measures is more effective than any single measure (*6*). Mitigation measures mandated through public policy can effectively increase social distancing (*7*), and wearing masks has prevented transmission of SARS-CoV-2 (*8*). In Arizona, decreases in daily COVID-19 cases were observed after widespread sustained community mitigation measures that promoted social distancing, limited large gatherings, paused operations of businesses where mask use and social distancing were difficult to maintain, mandated and enforced mask wearing, and promoted voluntary resident actions to stay at home and wear masks (when and where not mandated). The number of COVID-19 cases stabilized and began to decrease approximately 2 weeks after local officials began mandating mask wearing (throughout several counties and cities) and enhanced sanitation practices. Additional declines in case counts were associated with implementation of statewide limitations and closures sustained throughout July and extended into August.

The findings in this report are subject to at least four limitations. First, the relationship between mitigation measures and changes in case counts are temporal correlations and should not be interpreted to infer causality. Other factors that might have influenced the rate of change (e.g., travel restrictions, neighboring state mitigation measures, and individual choices to reduce movement before implementation of mandates) cannot be ruled out. Second, health centers run by tribal entities and federal health facilities (i.e., Indian Health Service, Veteran’s Administration, and Department of Defense) in the state are requested but not required to comply with state reporting rules. Many of these health centers and federal health facilities complied with reporting, but the completeness of reporting by these entities is unknown. Third, adherence to mitigation measures was not assessed, nor could the extent to which each individual measure affected the number of incident COVID-19 cases be established. Finally, Arizona might not be representative of other U.S. states, and community mitigation measures might have a different impact in more populous or densely populated states; thus, these findings are not necessarily generalizable to other settings.

Enhanced mitigation measures should be implemented by communities and persons to slow COVID-19 spread, particularly before a vaccine or therapeutic treatment becomes widely available. State, local, and tribal officials are best positioned to continually monitor data and collaborate to determine the level and types of enhanced mitigation required. Mitigation measures, including mask mandates, that are implemented and enforced statewide appear to have been effective in decreasing the spread of COVID-19 in Arizona.

**Summary**

**What is already known about this topic?**

Community mitigation measures can help slow the spread of COVID-19.

**What is added by this report?**

The number of COVID-19 cases in Arizona stabilized and then decreased after sustained implementation and enforcement of statewide and locally enhanced mitigation measures, beginning approximately 2 weeks after implementation and enforcement of mask mandates and enhanced sanitations practices began on June 17; further decreases were observed during July 13–August 7, after statewide limitations and closures of certain services and businesses.

**What are the implications for public health practice?**

Widespread implementation and enforcement of sustained community mitigation measures, including mask wearing, informed by state and local officials’ continual data monitoring and collaboration can help prevent transmission of SARS-CoV-2 and decrease the numbers of COVID-19 cases.

## Footnotes

* Mandates and ordinances varied and were county- and city-specific. Enforcement types included educating persons on the dangers of COVID-19 spread, issuing fines to persons and businesses who refused to comply with mandates, and loss of licenses for businesses not enforcing rules or mandates.

† https://cdn.ymaws.com/www.cste.org/resource/resmgr/ps/positionstatement2020/Interim-20-ID-02_COVID-19.pdf.

§ https://azdhs.gov/preparedness/epidemiology-disease-control/infectious-disease-services/index.php#medsis-faqs.

¶ https://www.azdhs.gov/preparedness/epidemiology-disease-control/infectious-disease-epidemiology/covid-19/dashboards/index.php.

** Based on guidance from ADHS, CDC, and the Department of Labor, and Occupational Safety and Health Administration to limit and mitigate the spread of COVID-19, including promoting healthy hygiene practices; and intensifying cleaning, disinfection and ventilation practices.

†† https://www.azdhs.gov/preparedness/epidemiology-disease-control/infectious-disease-epidemiology/index.php#novel-coronavirus-admin-orders; https://azgovernor.gov/executive-orders.
